# Selenite reduces algal reactive oxygen species accumulation and enhances algal resistance to a bacterial pathogen

**DOI:** 10.1093/ismeco/ycag126

**Published:** 2026-05-12

**Authors:** Roni Beiralas, Shira Ben-Asher, Einat Segev

**Affiliations:** Department of Plant and Environmental Sciences, Weizmann Institute of Science, Rehovot 7610001, Israel; Department of Plant and Environmental Sciences, Weizmann Institute of Science, Rehovot 7610001, Israel; Department of Plant and Environmental Sciences, Weizmann Institute of Science, Rehovot 7610001, Israel

**Keywords:** *Emiliania huxleyi*, *Phaeobacter inhibens*, selenite, selenium, oxidative stress, reactive oxygen species, antioxidants, algal protection, algal defense, bacterial pathogenicity

## Abstract

Oxidative stress arises when cells fail to maintain redox balance. Among marine microalgae, oxidative stress is a hallmark of interaction with pathogenic bacteria. In co-culture of *Emiliania huxleyi* algae with pathogenic bacteria of the species *Phaeobacter inhibens*, algal intracellular levels of reactive oxygen species (ROS) increase prior to bacterial-induced population collapse. Here, we tested whether antioxidant amendments can alter the pathogenic outcome. Screening several environmentally relevant antioxidants revealed that low-nanomolar levels of selenite [Se (IV)] prevented algal death in co-culture. Time-resolved ROS measurements showed that selenite-treated co-cultures maintained lower intracellular ROS levels during exponential growth, while bacterial growth dynamics were comparable with and without selenite. Together, these results show that selenite availability shifts the outcome of the *E. huxleyi*–*P. inhibens* interaction and link host oxidative stress to resilience in co-culture.

## Introduction

Algal blooms are vast oceanic events that are often terminated by a rapid demise [1]. The bloom collapse can be caused by various reasons, including viral infection and possibly pathogenic bacteria [2, 3]. In laboratory co-culture systems of algae and bacteria, bacterial pathogenicity toward algae has been well-documented over the past decade [3–8].

A recurring hallmark of bacterial pathogenesis in microalgae is the intracellular accumulation of reactive oxygen species (ROS), a surge that can precede or coincide with algal death. Importantly, this oxidative stress does not necessarily originate from bacterially produced ROS. Rather, bacterial pathogens often produce compounds that provoke the algal cells to generate ROS internally [3, 4, 8–11]. *Emiliania huxleyi* (taxonomically reassigned as *Gephyrocapsa huxleyi* [12]) is a globally abundant marine coccolithophore (Haptophyta) that forms extensive blooms and plays a major role in marine carbon cycling through both photosynthesis and calcification [13]. Its interactions with associated bacteria have been widely studied in the context of bloom dynamics and bacterial-driven mortality processes [5]. In the model co-culture system of *E. huxleyi* algae and pathogenic bacteria of the species *Phaeobacter inhibens*, abundant evidence supports that bacterial metabolites induce oxidative stress within the algal cells and promote algal population collapse [3, 9, 10, 14]. Importantly, previous work showed that in this algal–bacterial system, population collapse reflects algal death through a regulated, programmed cell death (PCD)-like process [3, 9]. Moreover, in these co-cultures, most algal cells accumulate ROS, and ROS quenching was shown to rescue the algal population from collapsing [9]. Transcriptomic data further show that genes involved in ROS detoxification are among the most strongly up-regulated algal transcripts during the *E. huxleyi*–*P. inhibens* interaction [3, 10]. Recently, a specific pathway of oxidative stress induction in algae was reported in the *E. huxleyi*-*P. inhibens* model system, demonstrating that algal-derived betaine stimulates bacterial H₂O₂ production, which in turn promotes algal death [14].

Oxidative stress is central in various algal–bacterial interactions; ROS accumulation is linked to apoptotic or apoptosis-like death in bloom-forming algae such as *Phaeocystis globosa* and *Alexandrium tamarense* when exposed to *Microbulbifer* or *Brevibacterium* bacterial pathogens [4, 8]. The bacterial pathogen *Shewanella sp. IRI-160* which causes the death of several dinoflagellate species, was shown to produce a compound that increases intra- and extracellular ROS in dying algae [11]. Together, these studies support the view that oxidative stress is a common feature of algal responses during interactions with antagonistic bacteria, although additional stressors likely contribute to algal mortality outcomes.

Beyond possibly promoting algal death, oxidative stress in microalgae can have broader ecological and economic consequences. Excess ROS promote the production of transparent exopolymer particles (TEP) and sticky mucilage [15–17]. TEP enhances cell–cell aggregation, accelerates vertical carbon export, and can accumulate as dense foams that clog coastal infrastructure [18–20]. Oxidative stress is also linked to the synthesis and release of potent algal toxins: in *Microcystis aeruginosa*, exogenous ROS addition promotes microcystin production [17], while in several dinoflagellate species, oxidative stress is linked to algal toxicity [21, 22]. Thus, oxidative stress can amplify the ecological and economic impacts of bloom events through mucilage formation and toxin release. These broader consequences motivate a closer characterization of oxidative-stress dynamics during microbial interactions, and the conditions that modulate them.

One way to probe the role of oxidative stress in algal–microbe interactions is to perturb redox conditions using exogenous antioxidants or micronutrients. In higher plants, the addition of redox-active compounds such as ascorbate and glutathione, as well as micronutrients such as selenium, can reduce ROS build-up and improve performance under stress [23, 24]. Microalgae likewise rely on conserved antioxidant networks that include ascorbate- and glutathione-based detoxification pathways, and these systems are dynamically regulated in response to oxidative stress [25, 26]. Testing exogenous antioxidant amendments in algal–bacterial co-cultures can therefore serve as an experimental approach to evaluate how host redox buffering influences interaction trajectories, and whether shifting oxidative-stress dynamics is sufficient to alter the outcome of antagonistic encounters.

In this study, we used the *E. huxleyi–P. inhibens* model system to test whether antioxidant amendment can alter the trajectory and outcome of bacteria-induced algal death. We screened a range of environmentally relevant antioxidants and found that low-nanomolar levels of selenite (Se (IV)) prevented algal death in co-culture with bacteria. Time-resolved experiments using a ROS reporter showed that selenite-treated co-cultures maintained reduced oxidative-stress levels in the algal population during the period that precedes algal collapse in the other treatments. Together, these results suggest that selenite can alter the trajectory of the *E. huxleyi*–*P. inhibens* interaction, with algal resilience correlating with reduced intracellular ROS in the algal population.

## Materials and methods

### Strains and growth conditions

The algal strain of *Emiliania huxleyi* CCMP2090 was purchased as an axenic culture from the National Center for Marine Algae and Microbiota (Bigelow Laboratory for Ocean Sciences, ME, USA). Algae were maintained in artificial seawater medium (ASW) prepared according to Sperfeld *et al.* [27] and supplemented with L1 nutrients (NaNO_3_, 882 μM; NaH_2_PO_4_·2H_2_O, 36.22 μM), L1 vitamins (thiamine HCl, 100 μg l^−1^; biotin, 0.5 μg l^−1^; vitamin B_12_, 0.5 μg l^−1^) and f/2 trace metals (Na_2_EDTA·2H_2_O, 4.36 mg l^−1^; FeCl_3_·6H_2_O, 3.15 mg l^−1^; MnCl_2_·4H_2_O, 178.1 μg l^−1^; ZnSO_4_·7H_2_O, 23 μg l^−1^; CoCl_2_·6H_2_O, 11.9 μg l^−1^; CuSO_4_·5H_2_O, 2.5 μg l^−1^; Na_2_MoO_4_·2H_2_O, 19.9 μg l^−1^). The prepared ASW was adjusted to pH −8. Stock solutions of nutrients, vitamins, and trace metals were purchased from the Bigelow Laboratory. Cultures were grown in sterile glass Erlenmeyer flasks (Flask volume: 250 ml; Media volume: 30 ml). Algae were grown in standing cultures in a growth room at 18°C under a light/dark cycle of 16/8 hr. Illumination intensity during the light period was provided by LED lighting at 130 μmol photons m^−2^ s^−1^. Absence of bacteria in axenic algal cultures was monitored weekly both by plating on ½ YTSS plates (containing yeast extract, 2 g l^−1^; tryptone, 1.25 g l^−1^; sea salts, 20 g l^−^1) and by bright-field microscopy (×40 objective).

The bacterial strain of *Phaeobacter inhibens* DSM 17395 was purchased from the German collection of microorganisms and cell cultures (DSMZ, Braunschweig, Germany). Bacteria were cultured in ½ YTSS medium, either in liquid culture or on agar plates, and incubated at 30°C with shaking at 130 rpm.

### Algal cell counts

Algal cell abundance was quantified using a CellStream CS-100496 flow cytometer (Merck, Darmstadt, Germany). Chlorophyll autofluorescence was excited with the 561 nm laser and collected in the 702/87 nm emission channel. Samples were acquired at a flow-rate setting of 14.64 μl/min, and 50 000 total events were recorded per sample. Algal events were identified by gating on forward scatter (FSC) together with chlorophyll autofluorescence. Gates were defined separately for each sampling day using an axenic *E. huxleyi* control acquired on the same day (e.g. the Day 7 gate was defined using the Day 7 axenic culture) and subsequently applied to all samples measured for that day.

### Algal–bacterial co-culturing

Co-cultures of *E. huxleyi* and *P. inhibens* were established as follows: algal cells from a late-exponential phase culture were quantified using flow cytometry as described above. An inoculum of 10^3^ algal cells was introduced into 30 ml of ASW medium supplemented with nutrients, vitamins, and trace metals. The time point of algal inoculation was designated “Day −4”. After four days of algal growth, *P. inhibens* bacteria were prepared for co-culturing. Bacteria were harvested from 48 h liquid culture in ½ YTSS medium and washed three times with ASW. Then, bacteria were diluted to an OD_600_ of 0.01 measured with Ultrospec 2100 *pro* spectrophotometer (Biochrom, UK) using a 1 cm path length polystyrene cuvette. This suspension was further diluted to 1:1000, and 20 μl was added to the algal cultures. The time point of bacterial addition into algal cultures was designated “Day 0”. Co-cultures were incubated in the same growth conditions as described for axenic algal cultures. Sampling days are indicated relative to the time of bacterial addition.

### Monitoring bacterial growth in co-cultures

Bacterial abundance in co-cultures was assessed at Days 0, 3, 7, 10, 14, and 17. Samples were serially diluted in ASW and plated on ½ YTSS agar plates. Plates were incubated for 48 h at 30°C. Colony-forming units (CFUs) were counted after incubation, and bacterial concentrations in the original samples were calculated accordingly.

### Intracellular reactive oxygen species measurements in algal cells

Intracellular ROS levels in algal cells were measured using the fluorescent dye 2′,7′-dichlorodihydrofluorescein diacetate (H_2_DCFDA; ThermoFisher), dissolved in DMSO. Fluorescence was detected using a CellStream CS-100496 flow cytometer, with excitation at 488 nm and emission collected at 528/46 nm. Algal cells were gated as described above for algal cell counts.

For each measurement, 400 μl of culture was sampled and split into two 200 μl sub-samples. One sub-sample served as a negative control and was treated with DMSO; the other was treated with 5 μM H_2_DCFDA. Samples were incubated in the dark for 30 min prior to measurement. Mean fluorescence intensity of the gated algal population was quantified using CellStreamAnalysis software. Background fluorescence (from the DMSO-treated control) was subtracted from the fluorescence of the H_2_DCFDA-treated sample to obtain the corrected intracellular ROS signal in the algal population.

### Antioxidants screen

Axenic *E. huxleyi* cultures and *E. huxleyi–P. inhibens* co-cultures were prepared as described above, with antioxidant amendments added either at the time of algal inoculation (Day −4) or on Day 7 of algal growth, as indicated. Selenium was supplied at 1 nM as selenous acid (H₂SeO₃; Sigma-Aldrich, 98%); throughout the manuscript we refer to this amendment as selenite, as at pH 8 in ASW Se(IV) is present predominantly as HSeO₃^−^/SeO₃^2−^. L-ascorbic acid (10 μM; Sigma-Aldrich, 99%) is referred to as L-ascorbate in the manuscript. α-tocopherol (10 μM; Sigma-Aldrich, ≥95%) and dimethylsulfoniopropionate (DMSP) (50 μM; sulfonium, (2-carboxyethyl)dimethyl-, chloride; Angene, 97%) were used as indicated. Stock solutions were prepared in ddH₂O, except α-tocopherol, which was dissolved in DMSO (dimethyl sulfoxide; MP Biomedicals). To control for potential solvent effects, a DMSO-only condition was included (See supplementary data). Algal and bacterial abundances were monitored over time as described above.

### Data processing and statistical analyses

**Figure 1:**
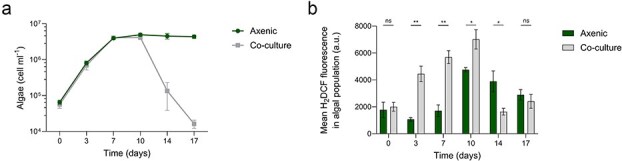
Algal growth and intracellular ROS in axenic cultures and co-cultures with bacteria. The growth of the algal strain *E. huxleyi* along 17 days cultured axenically or in co-culture with bacteria of the species *P. inhibens* (a). Mean fluorescence intensity of the probe 2′,7′-dichlorodihydrofluorescein diacetate (H_2_DCFDA) which indicates oxidative stress in *E. huxleyi* populations along 17 days of growth (b). Each data point consists of three biological replicates, error bars designate ± SD. Statistical significance was calculated using unpaired *t*-tests to compare algal abundance or H_2_DCFDA fluorescence between axenic cultures and co-cultures. *P*-values were adjusted for multiple testing using the Holm–Šídák method. ^*^P < .05, ^**^P < .005, ns—not significant. Exact *P*-values are provided in Tables S8 and S9.

**Figure 2:**
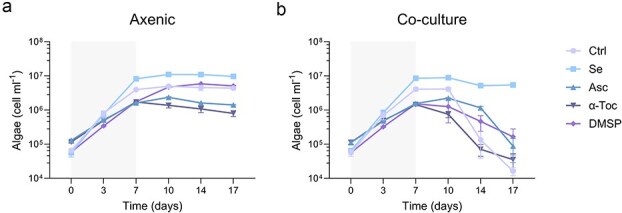
Algal growth in axenic cultures and co-cultures supplemented with different antioxidants. The growth of the algal strain *E. huxleyi* along 17 days either axenically (a) or with the bacterial species *P. inhibens* in co-culture (b). The cultures were either without supplementation (Ctrl) or with antioxidant amendments added during algal inoculation: Selenite (Se, 1 nM), L-Ascorbate (Asc, 10 μM), α-tocopherol (α-toc, 10 μM), and dimethylsulfoniopropionate (DMSP, 50 μM). Grey shading denotes the shared exponential-growth window (Days 0–7) used for within-stage comparisons in subsequent analyses (Fig. 3). Each data point consists of three biological replicates, error bars designate ± SD. Statistical significance was calculated using unpaired *t*-tests to compare algal abundance between untreated (Ctrl) and treated samples (Se/Asc/α-toc/DMSP). *P*-values were adjusted for multiple testing using the Holm–Šídák method. Exact *P*-values are provided in Table S8.

Data on algal and bacterial cell abundances and H₂DCFDA fluorescence were collected from three biological replicates for each condition and time point. Tables S1–S7 report the corresponding means ± SD. Statistical analyses were performed for algal abundance, bacterial abundance and H₂DCFDA fluorescence using GraphPad Prism v10.1.1 software. All statistical tests were performed using unpaired *t*-tests with a correction for multiple testing using the Holm–Šídák method. Comparisons were made between axenic and co-cultures (Fig. 1a and b) and between non-treated samples (Ctrl) and treated samples (Se, Asc, α-Toc, and DMSP) (Figs 2a and b;  S1; and S2a and b) at the same time point. Exact *P*-values are provided in Tables S8–S10.

## Results

### Algal oxidative stress increases during exponential growth in co-cultures with bacteria

To investigate oxidative stress in algae, we monitored axenic *E. huxleyi* CCMP2090 cultures grown either alone or in co-culture with *P. inhibens* DSM17395 (see Materials and Methods). Over a 17-day growth period, we tracked algal and bacterial cell numbers using flow cytometry and plating, respectively. To assess oxidative stress in algal cells, we measured intracellular ROS using the fluorescent probe 2′,7′-dichlorodihydrofluorescein diacetate (H_2_DCFDA; see Materials and methods) (Fig. 1). The fluorescence signal was detected via flow cytometry, which enabled us to quantify H_2_DCFDA-derived fluorescence within the gated algal population. Although ROS accumulation has been implicated in algal–bacterial antagonisms more broadly, this dataset provides a high-temporal-resolution characterization of ROS dynamics in this algal–bacterial system.

Algal death occurred in the co-cultures after Day 10, while the axenic algal cultures remained stationary and did not exhibit a similar demise (Fig. 1a). Axenic cultures exhibited stable levels of intracellular ROS during the exponential growth phase (Days 0–7), followed by a transient ROS peak around Day 10 that decreased by later time points (Fig. 1b). Algae in co-cultures, however, showed significantly increased ROS levels compared to axenic cultures during the exponential growth phase (Days 3–7) and on Day 10 (Fig. 1b). On Day 14, ROS levels in co-cultures were lower than in axenic cultures, likely because co-cultures post-demise contain a small surviving algal population (Fig. 1a), as was previously shown [3]. It is difficult to compare algal ROS levels between axenic cultures and co-cultures at this late time point since algae are in a different growth stage and algal cell concentration markedly differ. Notably, the elevated algal oxidative stress in the co-cultures compared with axenic cultures was significant on Days 3, 7, and 10 both prior to and at the onset of algal death. Interestingly, the most pronounced difference in algal oxidative stress between co-cultures and axenic cultures occurred on Day 3 and 7, before the onset of algal death (Fig. 1). In co-cultures, bacteria grew and reached the stationary phase around Day 10 (Fig. S1, Ctrl).

Together, these findings show that intracellular ROS levels increase early during co-culture and precede the major decrease in algal viability, consistent with elevated levels of oxidative stress prior to algal population collapse.

### Selenite prevents algal death in co-cultures with bacteria

Building on our observation that oxidative stress may play a role in bacterially mediated algal population collapse (Fig. 1), we next asked whether supplementation with exogenous antioxidants could alleviate algal decline by reducing oxidative stress. We intentionally selected a panel of antioxidant molecules that are naturally present in marine systems and/or produced by marine phytoplankton, and that span distinct chemical properties and cellular targets, including aqueous versus lipid phase and metabolite versus trace element cofactor. Specifically, we tested selenite [Se(IV)], a bioavailable selenium species that is taken up by *E. huxleyi* via a high affinity transport system and incorporated into selenoproteins involved in redox homeostasis [28, 29]; dimethylsulfoniopropionate (DMSP), a hallmark *E. huxleyi* metabolite that also functions as a stress protectant and can contribute to ROS scavenging [30]; and the vitamins L-ascorbate (vitamin C) and α-tocopherol (vitamin E), which are widely distributed in microalgae and represent complementary water soluble and lipid soluble antioxidant strategies [31, 32]. To choose relevant concentrations for our experiments, we aimed to approximate ecologically plausible exposures. We acknowledge that bulk seawater concentrations can substantially underestimate the levels microbes experience at the microscale around algal cells, known as the phycosphere [5, 33]. We supplemented selenite at 1 nM, which falls within the order of magnitude measured for dissolved selenite in seawater and coastal environments, and within the range discussed for seawater selenite availability [34, 35]. DMSP is typically detected in bulk seawater at nanomolar concentrations, but microscale hotspots around phytoplankton cells and in dense cultures can reach micromolar levels [5, 6, 36, 37]. Axenic stationary phase *E. huxleyi* cultures can produce up to approximately 70 μM DMSP, and 100 μM DMSP has been used to induce bacterial pathogenicity [6, 36]. We therefore used 50 μM DMSP as a conservative, submaximal concentration that remains within the upper micromolar range reported for dense *E. huxleyi* cultures while avoiding deliberate induction of bacterial virulence. Reported intracellular pools of the antioxidant vitamins L-ascorbate and α-tocopherol in microalgae span orders of magnitude (L-ascorbate: 0.06–18.79 mg per g dry weight; α-tocopherol: 0.01–6.32 mg per g dry weight), corresponding to approximate intracellular concentrations of 1 μM to 10 mM [38]. Because dissolved concentrations around cells are difficult to constrain and depend strongly on exudation and diffusion [5, 33], we supplemented 10 μM L-ascorbate and 10 μM α-tocopherol as conservative low micromolar additions that are well below the upper end of reported cellular pools.

Our results show that in axenic cultures, the tested antioxidants affected algal growth trajectories, although no population collapse was observed (Fig. 2a). Cultures amended with selenite reached higher final cell densities, whereas L-ascorbate and α-tocopherol resulted in lower cell densities relative to the untreated control (Fig. 2b). DMSP-amended cultures reached a similar final cell density as the control, but this level was reached later in the time course (Day 10 rather than Day 7). In co-cultures with the bacterial pathogen, the algal population collapsed in all treatments except selenite amendment (Fig. 2b). Selenite-treated co-cultures maintained high algal abundance throughout the experiment, showed only a modest decline at later time points without rapid collapse, and reached final densities comparable to selenite-treated axenic cultures (Fig. 2a and b). Importantly, during the antioxidant screen, the growth of *P. inhibens* was monitored to ensure that the observed mitigation of pathogenicity was not due to inhibition of bacterial growth (Fig. S1). Under all treatments, bacteria grew to comparable final densities. Notably, bacterial growth in co-cultures supplemented with selenite was comparable to bacterial growth under control conditions, although the typical algal death was not observed under selenite addition (Fig. 2b, Fig. S1).

To test whether the timing of antioxidant addition influences the co-culture outcome, we repeated the experiment but supplemented antioxidants once after 7 days of co-culturing rather than at algal inoculation (Fig. S2a). Under these conditions, selenite addition at Day 7 was sufficient to rescue the algal population and prevent demise, whereas all other treatments failed to alter the outcome and the algal population collapsed. Selenite also increased final algal cell densities even when supplied only at Day 7, suggesting that late supplementation remains beneficial. Notably, late DMSP addition was associated with an earlier algal decline relative to untreated co-cultures.

These findings show that selenite prevents bacterial-induced algal population collapse. Given its redox activity, we next asked whether selenite treatment is associated with altered algal oxidative-stress dynamics in co-culture.

### Selenite reduces algal oxidative stress during exponential growth

**Figure 3:**
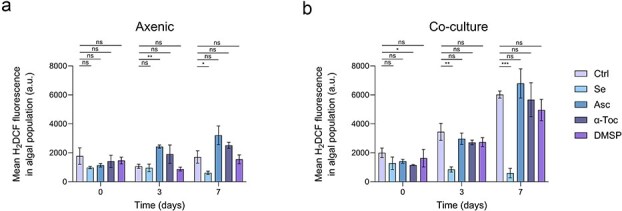
Algal intracellular ROS in axenic cultures and co-cultures supplemented with different antioxidants. Mean fluorescence intensity of the probe H_2_DCFDA in *E. huxleyi* populations grown axenically (a) or in co-culture with bacteria of the species *P. inhibens* (b) at Days 0, 3 and 7 (shared exponential growth window highlighted in grey in Fig. 2). The cultures were either without supplementation (Ctrl) or with antioxidant amendments added during algal inoculation: Selenite (Se, 1 nM), L-Ascorbate (Asc, 10 μM), α-tocopherol (α-Toc, 10 μM) and dimethylsulfoniopropionate (DMSP, 50 μM). Each data point consists of 3 biological replicates, error bars designate ± SD. Statistical significance was calculated using unpaired *t*-tests to compare H_2_DCFDA fluorescence between untreated (Ctrl) and treated samples (Se/Asc/α-Toc/DMSP). *P*-values were adjusted for multiple testing using the Holm–Šídák method. ^*^P < .05, ^**^P < .005, ^***^P < .0005, ns—not significant. Exact *P*-values are provided in Table S9.

In algal–bacterial co-cultures, elevated oxidative stress occurs prior to algal population collapse (Fig. 1). Therefore, we investigated whether the protective effect of selenite (Fig. 2b) is accompanied by altered intracellular ROS dynamics in *E. huxleyi* algae. To place the selenite phenotype in context and to help explain why other antioxidant treatments do not prevent collapse, we quantified intracellular ROS under all antioxidant treatments in both axenic cultures and co-cultures (Fig. 3). Because growth stages diverge after Day 7, with cultures entering stationary or decline phases, later time points are less directly comparable (Fig. 2). We therefore focus our comparisons on Days 0 to 7, during which cultures remain in a comparable exponential-growth window (Fig. 2, highlighted grey area) and differences in cell abundance that could complicate interpretation of fluorescence-based ROS measurements are minimized. Full ROS time courses are provided in the Supplementary Information (Fig. S3).

In axenic cultures (Fig. 3a), ROS levels were generally stable during exponential growth across treatments, with only modest, treatment-specific fluctuations. Selenite was associated with a significant reduction in ROS at Day 7, whereas L-ascorbate showed an increase in ROS at Day 3 relative to the control (Fig. 3a). At later time points, a transient ROS peak was observed between Days 10 and 14 in multiple conditions, suggesting that this peak reflects culture-stage physiology rather than a treatment-specific response (Fig. S3, green bars). In co-cultures, only selenite-treated algae maintained low and stable intracellular ROS during exponential growth (Fig. 3b). Relative to untreated controls, this reduction was already evident and significant by Days 3 and 7. All other treatments showed a gradual increase in ROS over time. At later time points, co-cultures exhibited a transient ROS peak between Days 10 and 14 (Fig. S3, grey bars), similar to axenic cultures (Fig. S3, green bars). When antioxidants were added to co-cultures at Day 7, only selenite significantly reduced ROS at Day 10, reducing the transient peak typically observed at this stage (Figs S2b and S3a). DMSP addition at Day 7 was also associated with lower ROS at Day 10, but this decrease was not statistically significant (Fig. S2b). Importantly, late DMSP addition accelerated algal collapse in co-culture (Fig. S2a), therefore the lower ROS signal at Day 10 may reflect measurements from a small surviving subpopulation.

Together, these results show that selenite treatment is associated with reduced algal intracellular ROS during exponential growth in co-culture (Fig. 3b), which is the phase that precedes algal collapse in untreated co-cultures (Fig. 2b). Importantly, selenite does not eliminate oxidative-stress signals entirely, as a transient ROS peak was still observed at later time points (Fig. S3a). This pattern is consistent with selenite primarily reducing sustained oxidative stress during growth and/or enhancing the ability of algae to tolerate transient oxidative challenges, rather than abolishing ROS signaling altogether.

## Discussion

### Elevated oxidative stress precedes algal population collapse in co-culture with bacteria

Our data show that oxidative stress is an early and reproducible signature of the pathogenic interaction between *P. inhibens* and *E. huxleyi*. Prior to algal demise in co-cultures, algal intracellular ROS rise to significantly higher levels than in axenic cultures (Fig. 1). This pattern is consistent with prior work in a different *E. huxleyi* strain [9]. The timing suggests that oxidative stress precedes major algal cell loss, likely contributing to the progression toward collapse and is not solely a late by-product of dying cells.

ROS are established upstream markers in regulated cell-death pathways in phytoplankton [39], including *E. huxleyi* during viral infection [2]. This suggests that ROS play a role in shaping the algal mortality trajectory. However, these findings do not exclude additional bacterial effects, such as nutrient or trace element competition, metabolic interference, or other stressors. These factors may act in parallel and ultimately drive algal population collapse.

### Selenite protects algae from bacterially induced death and reduces intracellular algal reactive oxygen species

Supplementing low-nanomolar levels of selenite prevented bacterially induced algal population collapse even though the bacterial population reached comparable densities in treated and untreated co-cultures (Fig. 2 and Fig. S1). This protection was observed both when selenite was supplied at algal inoculation and when it was introduced later, on Day 7, prior to the onset of collapse (Fig. S2). Consistent with this outcome, selenite-treated algae in co-cultures accumulated markedly less intracellular ROS during exponential growth compared to untreated co-cultures (Fig. 3b), linking protection with a reduced algal oxidative-stress signature during the period that precedes collapse in other treatments. Notably, a late transient ROS peak was still observed at later time points (Fig. S3a), and selenite addition at Day 7 reduced but did not abolish this Day-10 ROS peak (Fig. S2b), indicating that selenite can reduce oxidative levels without eliminating ROS dynamics altogether. Importantly, a similar late ROS peak was also observed in selenite-treated axenic cultures (Fig. S3a), suggesting that this transient increase may reflect an intrinsic, time-dependent physiological transition of the algal culture rather than a strictly bacteria-dependent oxidative burst.

A plausible explanation for the influence of selenite on culture trajectory and ROS dynamics is that selenite could strengthen endogenous antioxidant defenses. In *E. huxleyi*, selenite can serve as a bioavailable selenium source. Following uptake and assimilation, selenium can support selenoprotein biosynthesis, including glutathione peroxidases and thioredoxin reductases, which provide highly efficient catalytic antioxidant capacity [40]. *E. huxleyi* possesses an unusually large selenoproteome with multiple glutathione peroxidases and thioredoxin reductases [38, 40, 41]. Boosting this enzymatic arsenal could help maintain ROS below damaging levels and limit progression toward collapse. In addition, selenium has been linked to improved recovery from oxidative damage in some algal systems [42], although other studies report limited or no protective effects at low selenium levels [43], emphasizing that selenium effects can be context dependent.

At the same time, the association between selenite supplementation and reduced ROS does not necessarily imply that selenite protects *E. huxleyi* algae solely by lowering oxidative stress. Selenite is a biologically active micronutrient that can influence algal physiology through multiple processes beyond ROS readouts, including growth and metabolic state [28, 44]. Consistent with this, selenite supplementation enhanced algal growth in our experiments (Fig. 2, Fig. S2a) and previous studies have likewise reported that selenium supplementation can enhance growth in *E. huxleyi* [44, 45]. Moreover, selenite utilization in microalgae is not limited to incorporation as selenocysteine into selenoproteins alone; selenite can also enter other cellular pools via assimilation routes linked to sulfur metabolism and be incorporated into organic forms and/or nonspecifically into proteins [46–48]. Together, these observations suggest that selenite may reduce sustained stress during growth and/or increase the capacity of the algal population to tolerate transient stress episodes. Additional selenite-dependent physiological effects may also contribute to resilience in co-culture. Dissecting the relative contribution of these pathways will require targeted follow-up experiments that quantify selenite uptake and incorporation and directly assess the intracellular selenite fate.

### Non-selenite antioxidants do not suppress reactive oxygen species or prevent algal collapse in co-culture

Several factors may explain why L-ascorbate, DMSP, and α-tocopherol did not measurably lower intracellular ROS in *E. huxleyi* and did not rescue the algal population in co-culture (Fig. 2b, Fig. 3b, Fig. S2 and Fig.  S3b, c, and  d). Unlike selenite, which can be integrated into cellular metabolism and support endogenous redox buffering [28], exogenously added organic antioxidants may have limited access to intracellular sites of ROS production and/or may be rapidly oxidized, diluted, or metabolized, resulting in little sustained effect on the ROS signal [49]. In addition, compound-specific bioavailability is likely important: α-tocopherol must be taken up and incorporated into membranes to act at lipid-phase oxidative sites, which may be inefficient at the low concentrations used here [50]. DMSP, in contrast, is a highly dynamic metabolite in co-culture that can be rapidly transformed or consumed by bacteria, so its concentration and chemical fate may change over time [6, 51]. Consistent with this interpretation, delayed addition at Day 7 did not rescue co-cultures or reduce intracellular ROS for any treatment other than selenite, arguing against a simple timing explanation (Fig. S2).

Notably, compared to untreated controls, all antioxidant amendments except selenite were associated with reduced growth rates and lower final algal densities in co-culture (Fig. 2b). This suggests that these compounds may impose physiological costs or perturb cellular homeostasis rather than relieve stress. Such effects could reflect interference with nutrient uptake or metabolism, or indirect consequences mediated by bacterial responses to the added metabolites. For example, DMSP, particularly when added at a later time point, accelerated algal decline in co-culture (Fig. S2a) and has previously been shown to enhance bacterial virulence against *E. huxleyi* when supplied exogenously [6]. At present, we cannot distinguish between these possibilities. Future work could address these mechanisms by quantifying compound persistence and uptake and by testing whether these amendments alter bacterial physiology, secreted metabolites, or virulence programs.

### Host redox balance as an indicator and determinant of interaction outcomes

Host redox balance is a common axis along which microbes influence host health and interaction outcomes. In our model system, reducing oxidative stress, rather than suppressing the pathogen itself, appears sufficient to prevent algal population collapse, positioning algal intracellular ROS as an early indicator of interaction state and trajectory. In this study we show that intracellular ROS increases during exponential growth in co-culture and precedes population collapse (Fig. 1), whereas selenite maintains lower ROS over this window and prevents algal death (Fig. 2, Fig. 3, Fig. S2). Importantly, selenite does not measurably alter bacterial growth (Fig. S1), suggesting that the shift in outcome reflects altered host physiology rather than reduced pathogen abundance. Comparable redox signatures were reported in diverse algal–bacterial systems, where bacterial metabolites promote oxidative bursts that precede algal death [3, 4, 8–11]. Viruses also exploit a related vulnerability: viral infection of *E. huxleyi* leads to elevated algal ROS early in the replication cycle, and chemical ROS quenching impairs virion production [52]. In addition, viral infection triggers a transient antioxidant response in the algal host that is ultimately insufficient, and infected cells lyse [53]. Beyond marine systems, microbial metabolites and effectors can similarly drive host ROS accumulation in mammalian and plant interactions, with oxidative surges contributing to pathology [54–56]. Strengthening detoxification capacity, either endogenously or through nutrient supplementation, can reduce redox imbalance and alleviate disease severity [57, 58]. Together, these observations place the *E. huxleyi*-*P. inhibens* interaction within a broader spectrum of redox-mediated antagonisms and identify selenite availability as an environmental condition that can shift interaction outcomes.

### Environmental outlook

In the marine environment, dissolved selenium occurs at low concentrations and can vary substantially across regions and over time due to differences in external inputs including dust deposition, coastal and runoff influences, and biogeochemical cycling that affects selenium speciation and bioavailability [59]. Such variability, particularly at the scale of microenvironments around cells and within blooms, could influence the physiological state of *E. huxleyi* and, consequently, its susceptibility to oxidative stress and pathogen-driven population collapse. Our finding that low-dose selenite supplementation (1 nM) alters the co-culture outcome motivates testing whether natural selenium variability correlates with infection dynamics and bloom resilience in the field. A tractable next step would be controlled mesocosm experiments in which environmentally realistic selenium amendments are applied to natural microbial communities while tracking *E. huxleyi* abundance, oxidative stress markers, and the composition and activity of associated bacteria over time. Such approaches would help establish whether selenium availability is a predictive factor for algal–bacterial interaction outcomes in situ.

## Supplementary Material

Supplemental_data_2_ycag126

## Data Availability

Data from this study is available from the corresponding author upon reasonable request.
