# Supplementary material for: Selenite reduces algal reactive oxygen species accumulation and enhances algal resistance to a bacterial pathogen
Source: ISME Commun. 2026 May 12;6(1):ycag126. doi: 10.1093/ismeco/ycag126 (PMC13235717; doi:10.1093/ismeco/ycag126)
Supplement: Supplemental_data_2_ycag126 [file supplemental_data_2_ycag126.pdf]

Supplemental data

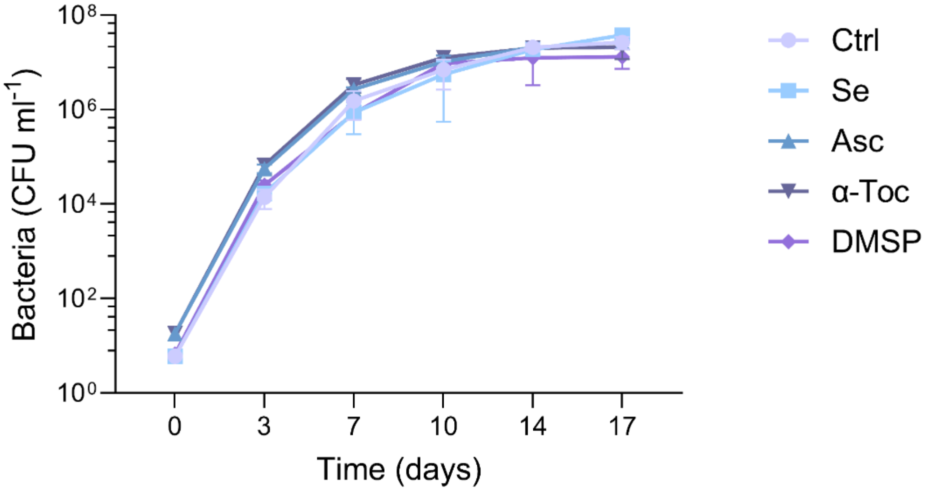

Figure S1. Bacterial growth in algal-bacterial co-cultures supplemented with different antioxidants. The growth of *P. inhibens* co-cultured with the algal strain *E. huxleyi* along 17 days. The co-cultures were either without supplementation (Ctrl) or with antioxidant amendments added at algal inoculum: Selenite (Se, 1 nM), L-Ascorbate (Asc, 10 µM), α-tocopherol (α-Toc, 10 µM) and dimethylsulfoniopropionate (DMSP, 50 µM). Each data point consists of 3 biological replicates, error bars designate ± SD. Statistical significance was calculated using unpaired t-tests to compare bacterial abundance between untreated (Ctrl) and treated samples (Se/Asc/α-Toc/DMSP). *p*-values were adjusted for multiple testing using the Holm-Šídák method. Exact *p*-values are provided in tables S10.

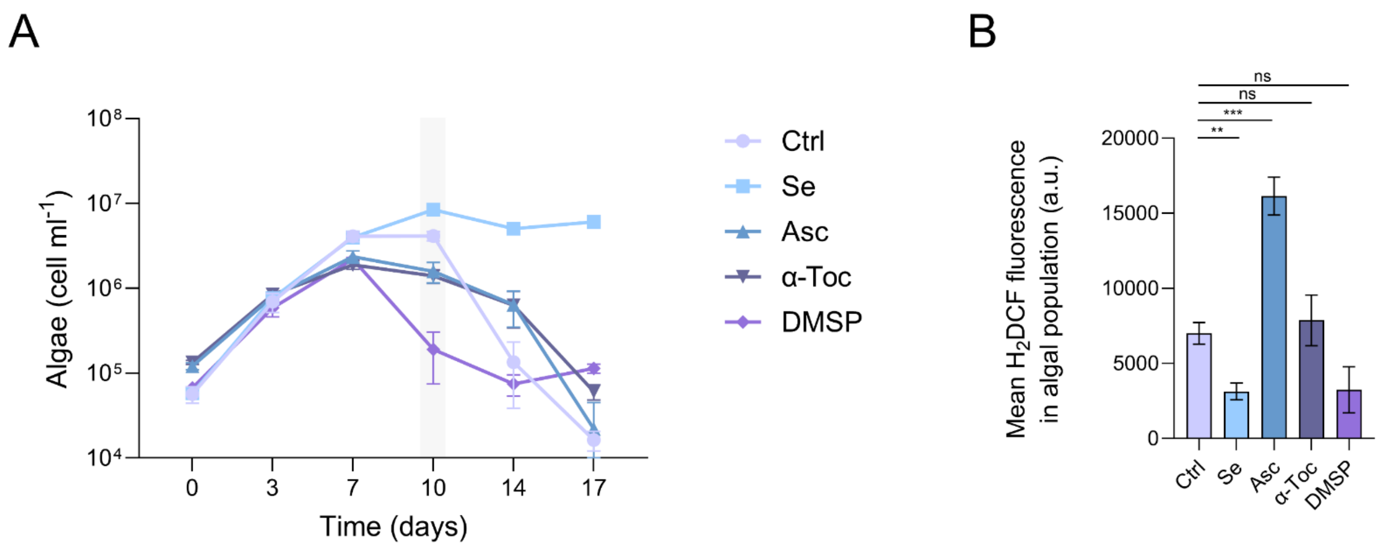

Figure S2. Algal growth and intracellular ROS in co-cultures supplemented with different antioxidants on day 7. Algal growth along 17 days in algal-bacterial co-culture **(A)**. Mean fluorescence intensity of the probe H_2DCFDA in algal populations on day 10 of algal growth (highlighted in grey in panel A) **(B)**. The co-cultures were either without supplementation (Ctrl) or with antioxidant amendments added at day 7 of algal growth: Selenite (Se, 1 nM), L-Ascorbate (Asc, 10 µM), α-tocopherol (α-Toc, 10 µM) and dimethylsulfoniopropionate (DMSP, 50 µM). Each data point consists of 3 biological replicates, error bars designate ± SD. Statistical significance was calculated using unpaired t-tests to compare algal abundance or H_2DCFDA fluorescence between untreated (Ctrl) and treated samples (Se/Asc/α-Toc/DMSP). *p*-values were adjusted for multiple testing using the Holm-Šídák method. ** $p < 0.005$, *** $p < 0.0005$, ns- not significant. Exact *p*-values are provided in tables S8 and S9.

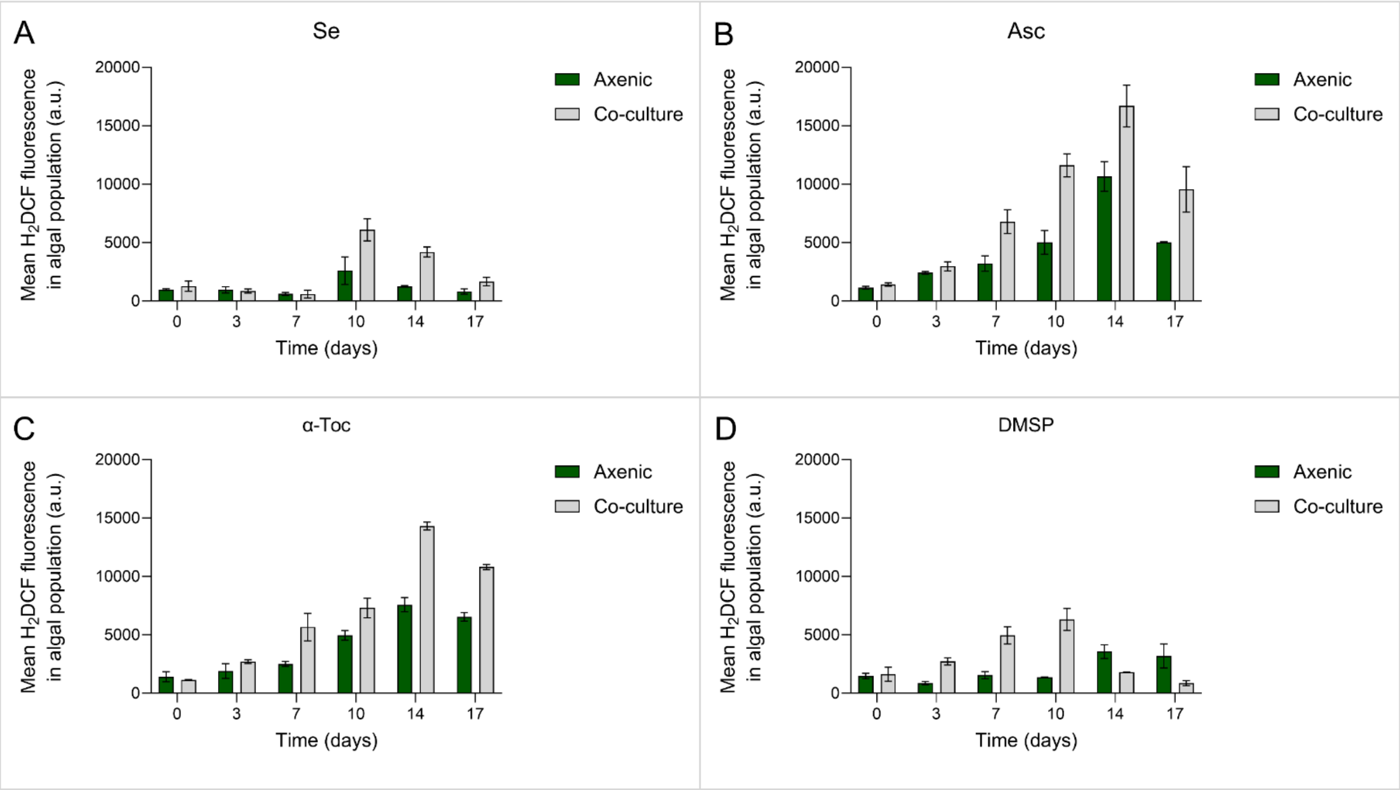

Figure S3. Intracellular ROS in axenic algal cultures and algal-bacterial co-cultures supplemented with different antioxidants along 17 days. Mean fluorescence intensity of the probe H_2DCFDA in axenic algal cultures (green bars) and co-cultures (grey bars) along 17 days of growth (corresponding to Fig. 2). The co-cultures were amended with antioxidants at algal inoculation: Selenite (Se, 1 nM) **(A)**, L-Ascorbate (Asc, 10 μM) **(B)**, α-tocopherol (α-Toc, 10 μM) **(C)** and dimethylsulfoniopropionate (DMSP, 50 μM) **(D)**. Each data point consists of 3 biological replicates, error bars designate ± SD.

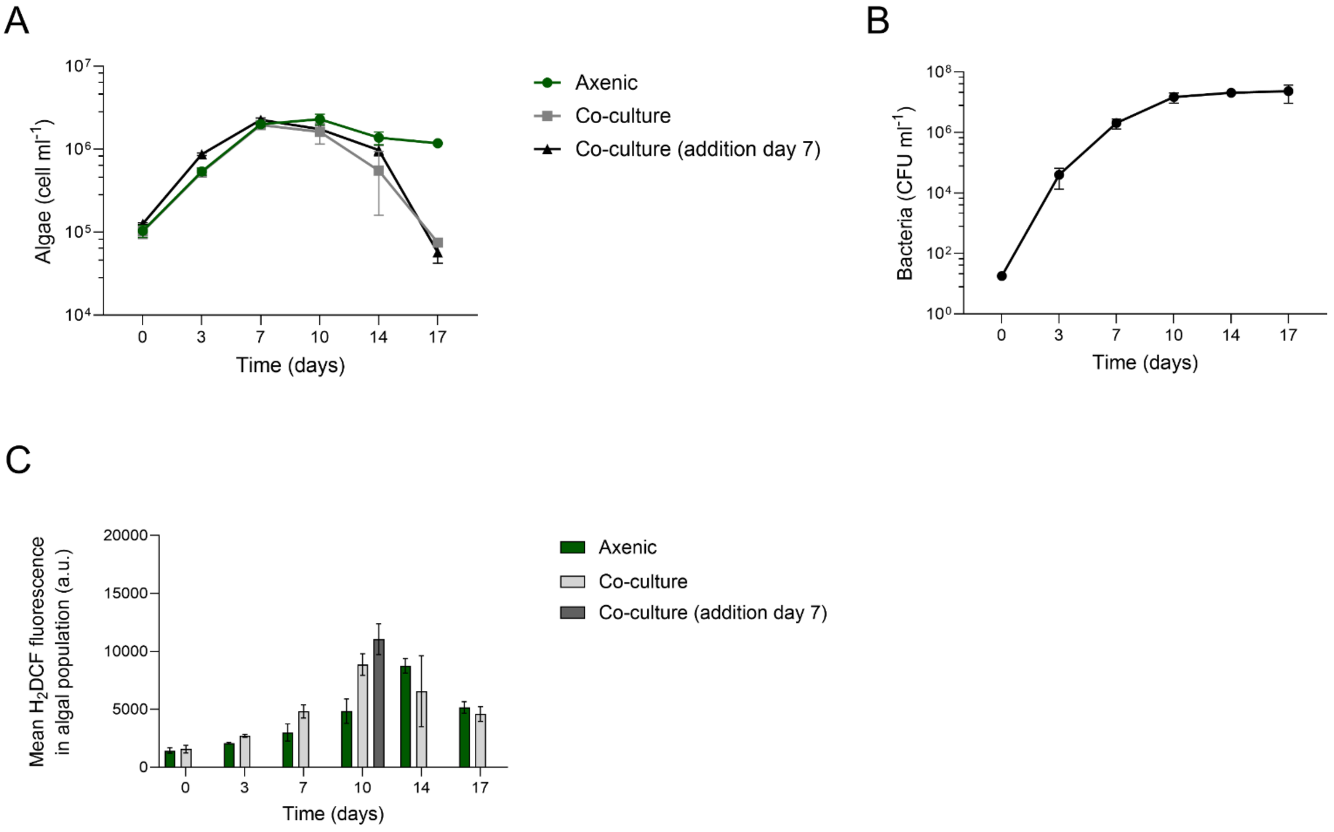

Figure S4. Algal growth, bacterial growth and intracellular ROS in axenic cultures and co-cultures supplemented with DMSO. Algal growth in axenic cultures and in algal-bacterial co-cultures that were supplemented with dimethyl sulfoxide (DMSO) either on algal inoculation (green line and light grey line) or on day 7 of algal growth (black line) **(A)**. Bacterial growth in co-cultures **(B)**. Mean fluorescence intensity of the probe H_2DCFDA in algal populations along 17 days of growth in cultures supplemented with DMSO during algal inoculation (green bar and light grey bar). In a parallel experiment, DMSO was supplemented on day 7 and H_2DCFDA fluorescence was measured on day 10 (dark grey bar) **(C)**. Each data point consists of 3 biological replicates, error bars designate ± SD.

	Ctrl mean	Ctrl SD	Se mean	Se SD	Asc mean	Asc SD	α-Toc mean	α-Toc SD	DMSP mean	DMSP SD	DMSO mean	DMSO SD
**0**	65409.74	7889.18	56347.99	14086.64	129082.4	10790.14	115743.3	9331.236	59148.33	872.994	103622.1	17681.53
**3**	809813.1	103897.4	756462.1	190780.3	518798.7	24491.18	493676.4	59891.43	343476.1	10816.36	537704.8	53793.24
**7**	3966699	156635.9	8225611	389528.1	1615071	72345.29	1719419	50600.79	1761561	179453.1	1998526	127319.3
**10**	4953745	490516.4	10998720	256215.4	2360531	109095.1	1369925	219855.4	4712947	75451.17	2298980	337284.1
**14**	4537743	783079.7	10930074	648890.8	1613060	21418.22	1077677	242167	5883054	79194.38	1374268	242664.2
**17**	4378377	479756.4	9646912	321179.9	1409809	53765.92	798775.6	159340.9	5053311	233743.4	1179528	10399.4

Table S1. Mean algal abundance (cell ml^{-1}) ± SD in axenic cultures supplemented with antioxidants at algal inoculation (day -4). Rows indicate sampling day, and columns indicate treatment. Means were calculated from three biological replicates. Ctrl, no antioxidant amendment; Se, selenite; Asc, L-ascorbate; α-Toc, α-tocopherol; DMSP, dimethylsulfoniopropionate; DMSO, dimethyl sulfoxide.

	Ctrl mean	Ctrl SD	Se mean	Se SD	Asc mean	Asc SD	α-Toc mean	α-Toc SD	DMSP mean	DMSP SD	DMSO mean	DMSO SD
0	56638.71	12526.91	64928.72	6370.56	111464.4	16105.15	113393.8	14539.76	57102.84	1783.755	102002.7	18191.09
3	692986.4	175062.6	852388.2	86395.35	479072.8	16164.54	501023.9	16116.5	324357.2	13132.92	526482.4	64279.86
7	4084047	555454.4	8573455	622390.8	1555160	44850.26	1449406	131446.6	1521606	109955.8	1958252	85593.38
10	4129753	511655.3	8888187	1277123	2234202	74836.45	757558.3	155016.9	1263053	842310.3	1612710	463299.1
14	134767.6	96421.11	5188837	448013.8	1160320	125787	70891.12	26700.78	464625.7	226268.7	549652.4	389560.6
17	16255.73	4204.193	5452415	448247.7	87857.2	59165.98	35393.55	17957.62	165204.9	116565.5	74831.05	7041.95

Table S2. Mean algal abundance (cell ml^{-1}) ± SD in co-cultures supplemented with antioxidants at algal inoculation (day -4). Rows indicate sampling day, and columns indicate treatment. Means were calculated from three biological replicates. Ctrl, no antioxidant amendment; Se, selenite; Asc, L-ascorbate; α-Toc, α-tocopherol; DMSP, dimethylsulfoniopropionate; DMSO, dimethyl sulfoxide.

	Ctrl mean	Ctrl SD	Se mean	Se SD	Asc mean	Asc SD	α-Toc mean	α-Toc SD	DMSP mean	DMSP SD	DMSO mean	DMSO SD
0	56638.71	12526.91	58418.96	7877.935	120780	11275.19	133658	7587.481	66595.02	1385.123	126477.5	3171.891
3	692986.4	175062.6	762073.5	74057.88	796372.5	105994.9	839296.5	33998.69	586449.7	127269.7	868478.5	29287.33
7	4084047	555454.5	3971455	265151	2361912	392063	1890307	219026.3	2199597	93816.1	2261819	116533.7
10	4129753	511654.9	8429336	1112776	1583425	431064.8	1392331	251373.4	189997.2	115668.9	1742143	204633.5
14	134767.6	96421.12	5039808	446915.7	636689.4	289255.9	627497.2	286966.9	74171.39	20866.24	974409.8	146222
17	16255.73	4204.193	6059996	614939.2	22006.64	22964.88	60436.26	12300.23	113582.5	13991.52	57171.33	15176.9

Table S3. Mean algal abundance (cell ml^{-1}) ± SD in co-cultures supplemented with antioxidants during algal growth (day 7). Rows indicate sampling day, and columns indicate treatment. Means were calculated from three biological replicates. Ctrl, no antioxidant amendment; Se, selenite; Asc, L-ascorbate; α-Toc, α-tocopherol; DMSP, dimethylsulfoniopropionate; DMSO, dimethyl sulfoxide.

	Ctrl mean	Ctrl SD	Se mean	Se SD	Asc mean	Asc SD	α-Toc mean	α-Toc SD	DMSP mean	DMSP SD	DMSO mean	DMSO SD
0	1775.133	570.0528	977.3667	76.84272	1140.133	123.2064	1414.067	423.7335	1468	232.4967	1449.867	235.043
3	1071.9	139.6159	961.8667	262.9632	2429.167	112.9876	1911.567	634.1479	876.2	133.5018	2084.1	71.33884
7	1711.867	431.4363	616.5	119.508	3210.267	644.1181	2515.133	209.301	1550.5	306.1772	3008.367	728.2639
10	4757.467	158.4225	2604.167	1174.916	5022.033	1013.331	4952.4	415.7701	1380.45	6.717514	4846.133	1048.188
14	3895.067	777.261	1258.7	67.02261	10655.67	1271.834	7594.067	601.6779	3567.3	587.4643	8750.667	620.5771
17	2894.633	388.6304	817.1667	216.6191	5027.433	53.45281	6551.667	356.7064	3190	1028.558	5161.733	497.3007

Table S4. Mean H_2DCFDA fluorescence (a.u.) in axenic algal populations ± SD following antioxidant supplementation at algal inoculation (day −4). Rows indicate sampling day, and columns indicate treatment. Means were calculated from three biological replicates. Ctrl, no antioxidant amendment; Se, selenite; Asc, L-ascorbate; α-Toc, α-tocopherol; DMSP, dimethylsulfoniopropionate; DMSO, dimethyl sulfoxide.

	Ctrl mean	Ctrl SD	Se mean	Se SD	Asc mean	Asc SD	α-Toc mean	α-Toc SD	DMSP mean	DMSP SD	DMSO mean	DMSO SD
0	2002.567	324.2988	1275.767	433.3129	1419.6	140.0631	1157.9	35.14612	1630.7	605.9905	1567.233	326.3206
3	4446.9	579.0837	852.6	180.0447	2970	390.914	2718.467	159.0357	2738.15	311.622	2714.3	129.2254
7	5690.9	483.0916	594.2	326.4149	6793.767	1012.251	5669.533	1174.35	4957.5	740.3408	4829.367	566.2615
10	7015.367	716.935	6100.3	936.2644	11606.77	986.1246	7302.233	842.068	6322.45	935.1487	8866.233	926.9693
14	1639.533	251.4319	4201.6	430.5956	16696.7	1785.137	14325.4	329.8437	1811	14.84924	6560.9	3056.41
17	2407	514.3714	1664.8	358.0122	9555.967	1946.608	10802.37	216.8832	879.45	215.8797	4590.8	638.7681

Table S5. Mean H_2DCFDA fluorescence (a.u.) in algal populations in co-cultures ± SD following antioxidant supplementation at algal inoculation (day −4). Rows indicate sampling day, and columns indicate treatment. Means were calculated from three biological replicates. Ctrl, no antioxidant amendment; Se, selenite; Asc, L-ascorbate; α-Toc, α-tocopherol; DMSP, dimethylsulfoniopropionate; DMSO, dimethyl sulfoxide.

	Ctrl mean	Ctrl SD	Se mean	Se SD	Asc mean	Asc SD	α-Toc mean	α-Toc SD	DMSP mean	DMSP SD	DMSO mean	DMSO SD
10	7015.367	716.935	3129.3	555.0515	16149.63	1266.226	7871.6	1694.212	3241.9	1533.573	11055.07	1325

Table S6. Mean H_2DCFDA fluorescence (a.u.) on day 10 in algal populations in co-cultures ± SD following antioxidant supplementation during algal growth (day 7). Rows indicate sampling day, and columns indicate treatment. Means were calculated from three biological replicates. Ctrl, no antioxidant amendment; Se, selenite; Asc, L-ascorbate; α-Toc, α-tocopherol; DMSP, dimethylsulfoniopropionate; DMSO, dimethyl sulfoxide.

	Ctrl mean	Ctrl SD	Se mean	Se SD	Asc mean	Asc SD	α-Toc mean	α-Toc SD	DMSP mean	DMSP SD	DMSO mean	DMSO SD
0	6	0	6	0	18	0	18	0	6.666667	0	18	0
3	14000	6082.763	16666.67	4618.802	56666.67	11547.01	66666.67	25166.11	25000	7071.068	40000	26457.51
7	1533333	929157.3	866666.7	568624.1	2700000	264575.1	3333333	1069268	825000	176776.7	2066667	750555.3
10	7000000	4358899	5500000	4949747	10333333	3511885	12666667	1527525	9500000	707106.8	15000000	5567764
14	20666667	3785939	18666667	2081666	20666667	4163332	20000000	5567764	12500000	9192388	20666667	577350.3
17	26666667	7637626	37666667	11503623	26333333	577350.3	21000000	9165151	13000000	5656854	23333333	14047538

Table S7. Mean bacterial abundance (CFU ml^{-1}) in co-culture with algae ± SD following antioxidant supplementation at algal inoculation (day −4). Rows indicate sampling day, and columns indicate treatment. Means were calculated from three biological replicates. Ctrl, no antioxidant amendment; Se, selenite; Asc, L-ascorbate; α-Toc, α-tocopherol; DMSP, dimethylsulfoniopropionate; DMSO, dimethyl sulfoxide.

	Fig. 1A	Fig. 2A				Fig. 2B				Fig. S2A			
	Axenic vs. Co-culture	Ctrl vs. Se	Ctrl vs. Asc	Ctrl vs. α-Toc	Ctrl vs. DMSP	Ctrl vs. Se	Ctrl vs. Asc	Ctrl vs. α-Toc	Ctrl vs. DMSP	Ctrl vs. Se	Ctrl vs. Asc	Ctrl vs. α-Toc	Ctrl vs. DMSP
0	ns	ns	0.003527	0.005574	ns	ns	0.028617	0.027222	ns	ns	0.016344	0.004825	ns
3	ns	ns	0.009158	0.010292	0.007510	ns	ns	ns	ns	ns	ns	ns	ns
7	ns	0.000308	0.000115	0.000114	0.000531	0.002946	0.007058	0.006619	0.008554	ns	0.046402	0.012445	0.017527
10	ns	0.000276	0.003461	0.001284	ns	0.011669	0.012550	0.002389	0.035930	0.014708	0.016344	0.005695	0.001209
14	0.003201	0.001212	0.005883	0.005574	ns	0.000221	0.002163	ns	ns	0.000296	ns	ns	ns
17	0.00057	0.000374	0.002199	0.001268	ns	0.000182	ns	ns	ns	0.000349	ns	0.012445	0.001610

Table S8. *p*-values for statistical tests performed on algal abundance. The top two rows indicate the corresponding figure and the specific comparison tested. Statistics were performed using unpaired t-tests for the indicated pairwise comparisons. Multiple testing correction using the Holm-Šídák method was applied, and *p*-values were adjusted accordingly. Color code: blue, $p < 0.05$; green, $p < 0.005$; orange, $p < 0.0005$; white, not significant (ns; $p \geq 0.05$).

	Fig. 1B	Fig. 3A				Fig. 3B				Fig. S2B			
	Axenic vs. Co-culture	Ctrl vs. Se	Ctrl vs. Asc	Ctrl vs. α-Toc	Ctrl vs. DMSP	Ctrl vs. Se	Ctrl vs. Asc	Ctrl vs. α-Toc	Ctrl vs. DMSP	Ctrl vs. Se	Ctrl vs. Asc	Ctrl vs. α-Toc	Ctrl vs. DMSP
0	ns	ns	ns	ns	ns	ns	ns	0.032484	ns	-	-	-	-
3	0.003019	ns	0.00059	ns	ns	0.003791	ns	ns	ns	-	-	-	-
7	0.002647	0.03933	ns	ns	ns	0.000063	ns	ns	ns	-	-	-	-
10	0.023705	-	-	-	-	-	-	-	-	0.001758	0.000406	ns	ns
14	0.026054	-	-	-	-	-	-	-	-	-	-	-	-
17	ns	-	-	-	-	-	-	-	-	-	-	-	-

Table S9. *p*-values for statistical tests performed on H_2DCFDA fluorescence in algal population. The top two rows indicate the corresponding figure and the specific comparison tested. Statistics were performed using unpaired t-tests for the indicated pairwise comparisons. Multiple testing correction using the Holm-Šídák method was applied, and *p*-values were adjusted accordingly. Color code: blue, $p < 0.05$; green, $p < 0.005$; orange, $p < 0.0005$; white, not significant (ns; $p \geq 0.05$).

	Fig. S1			
	Ctrl vs. Se	Ctrl vs. Asc	Ctrl vs. α-Toc	Ctrl vs. DMSP
0	ns	ns	ns	ns
3	ns	0.023749	ns	ns
7	ns	ns	ns	ns
10	ns	ns	ns	ns
14	ns	ns	ns	ns
17	ns	ns	ns	ns

Table S10. *p*-values for statistical tests performed on bacterial abundance. The top two rows indicate the corresponding figure and the specific comparison tested. Statistics were performed using unpaired t-tests for the indicated pairwise comparisons. Multiple testing correction using the Holm-Šídák method was applied, and *p*-values were adjusted accordingly. Color code: blue, $p < 0.05$; green, $p < 0.005$; orange, $p < 0.0005$; white, not significant (ns; $p \geq 0.05$).
